# The Use of Closed Incision Negative Pressure Therapy Immediately After Total Ankle Arthroplasty Surgeries

**DOI:** 10.7759/cureus.8606

**Published:** 2020-06-13

**Authors:** Alexandra Sidorski, Gregory Lundeen

**Affiliations:** 1 Wound Care, Renown Health, Reno, USA; 2 Foot and Ankle, Reno Orthopaedic Clinic, Reno, USA

**Keywords:** prevena, cinpt, total ankle arthroplasty, wound complication

## Abstract

Introduction

Total ankle arthroplasty (TAA) has become a common procedure in the treatment of end-stage ankle arthritis. Most prostheses utilize an anterior ankle approach, which has been shown to have incisional complication rates of up to 28%, including dehiscence and infection. Wounds in this area can be catastrophic to patient outcomes. Preventing incisional wounds would significantly benefit the patient. The purpose of this study was to evaluate the effect of closed incision negative pressure therapy (ciNPT) in reducing incisional dehiscence and surgical site infection (SSI) after TAA.

Materials and methods

A retrospective chart review that was approved by the Institutional Review Board (IRB) was performed on patients undergoing TAA. Inclusion criteria were patients undergoing TAA with an anterior incision and ciNPT placed immediately in the operating room. Comorbidities associated with increased wound complications were recorded. Identification of any incisional dehiscence, infections, or deviations from normal postoperative recovery attributed to the former was also recorded.

Results

Twenty-eight patients met the inclusion criteria. The average age of the patients at the time of surgery was 68 years. Comorbidities associated with compromised healing were obesity (45%), current or former smoking (45%), diabetes (3.5%), and rheumatoid arthritis (7%). There were no postoperative wound complications (100% incisional healing). No patient required any wound-care intervention or had an SSI. None of the patients had any delay in the normal postoperative course.

Conclusion

Avoiding wound complications in TAA patients is critical to the success of the procedure. This retrospective case series demonstrated 100% healing with the utilization of the ciNPT in both normal and high-risk patients with decreased healing potential. Our results showed a substantial decrease in wound complications and SSIs compared to historical reports. We recommend ciNPT for all TAA procedures utilizing an anterior incision to decrease the risk for wound complications and SSIs.

## Introduction

End-stage ankle arthrosis secondary to idiopathic and inflammatory etiologies may cause chronic disability and affect the patient’s quality of life. Total ankle arthroplasty (TAA) has become the primary treatment method for end-stage ankle arthritis. The procedure has been shown to improve function and increase the quality of life [1]. Increased patient demand for the procedure and improved cost-effectiveness will result in more TAA surgeries. A study by Matsumoto et al. has reported that TAA use has increased six-fold compared to previously reported numbers in 2010 [2]. As surgical techniques improve and the demand for the procedure increase, TAAs will likely be expanded to meet the surgical needs of more medically complex patients.

Although reductions in complications have improved the outcomes, wound healing and surgical site infections (SSIs) still remain a concern. Incidence of periprosthetic infection and wound complications after a TAA may result in long-term negative outcomes such as the need for revision arthroplasty, or even a below-knee amputation as the most severe result [2,3]. Wound-healing complications including delayed healing, suture-site infections, and local skin necrosis were present in 8% of patients according to a 2010 study, with a range of 0-14.7% for superficial infections [4]. In the same study, deep infections were observed in 0.8% of patients [4]. A more recent study in 2013 calculated the incidence of superficial infections seen to be 2.4% and deep infections to be 1.1% [5]. TAAs account for approximately 20% of elective ankle procedures that require intervention by plastic surgery [6].

Patient selection is important in preventing wound complications and infections. Characteristics such as advanced age, diabetes, smoking, rheumatoid arthritis, and obesity are all associated with incisional complications after TAA [3]. The incidence of wound complications may be reduced by paying close attention to surgical technique during closure and taking additional measures to minimize wound tension and edema [6]. Hematoma formation is a more common occurrence as more and more patients are using anticoagulation therapies. Hematomas increase the risk for SSIs due to dehiscence and tissue damage through excessive pressure [7]. The selection of certain dressings used post-surgery may also reduce complications during healing and improve patient outcomes. Dressings applied to a closed surgical incision act as a barrier to contamination from the environment and absorb fluid while providing a moist and more controlled environment to facilitate healing.

Negative pressure wound therapy (NPWT) was introduced in the United States in 1997 [8]. This technology helps to maintain a moist environment, optimizes blood flow, and promotes wound closure by affecting wound remodeling while also reducing the rates of infection. Other advantages include possible cost savings with the decreased need for dressing changes and other therapies if significant wound complications such as infection or dehiscence occur over the incision site [8]. Closed incision negative pressure therapy (ciNPT) facilitates fluid removal and acts as a barrier to contamination and infection. A meta-analysis showed a 50% reduction in SSIs in patients treated with ciNPT, as well as decreased scar formation [9,10]. These are important considerations in mitigating the possible complications of TAA surgery, particularly in high-risk patient populations [9].

Matsumoto and Parekh evaluated ciNPT in TAA patients using PICO single-use NPWT (Smith & Nephew, Memphis, TN). Their study demonstrated decreased wound problems compared to the control group that consisted of patients using conventional nonadherent gauze dressing (3% versus 24%, respectively) [11]. PREVENA Incision Management System (Acelity, San Antonio, TX) has been evaluated in hip and knee arthroplasty with decreases in SSIs and deep infections observed compared to controls [7]. There are differences between PREVENA and PICO, which may have an influence on clinical outcomes including negative pressure applied, surface area, and dressing composition. To our knowledge, there are no data currently available on the evaluation of PREVENA applied to TAA anterior incisions. The objective of our study was to evaluate the wound healing outcomes of a diverse patient population undergoing TAA treated with PREVENA and compare these results to historical reports. We hypothesize that PREVENA will demonstrate high rates of anterior incision healing in normal and high-risk patients compared to the rates of wound complications reported in the literature.

## Materials and methods

Surgical and PREVENA technique

**Figure 1:**
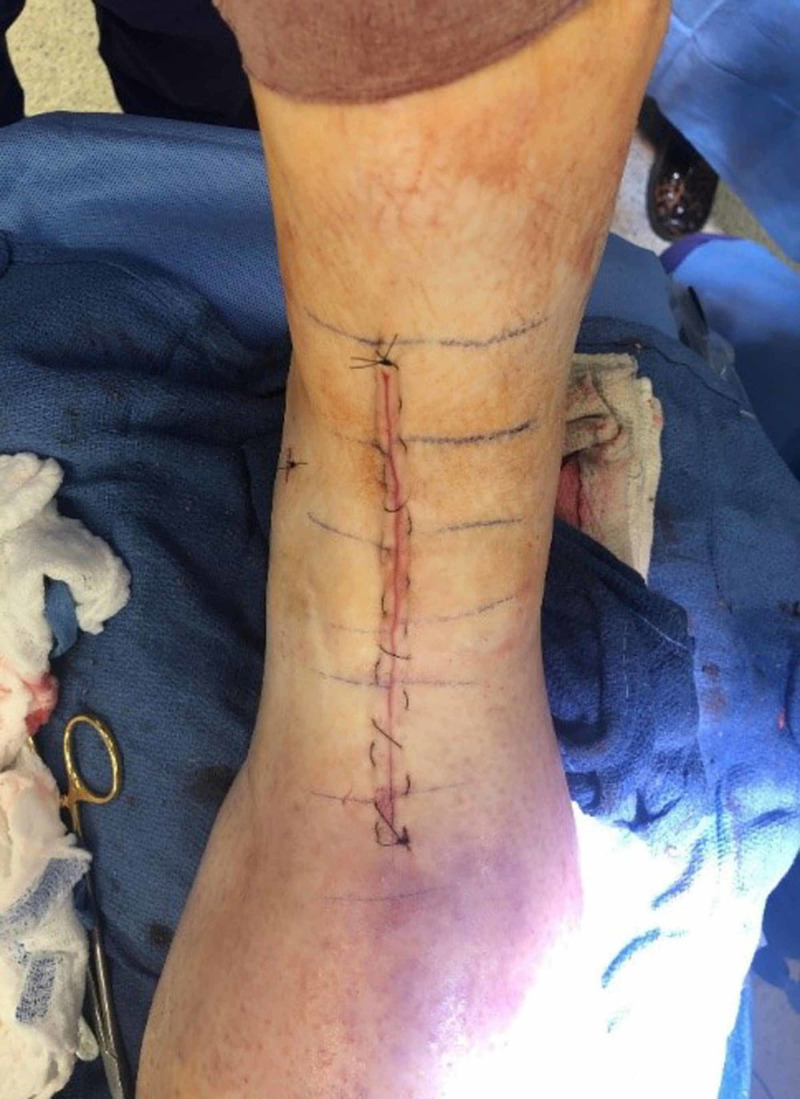
Total ankle arthroplasty anterior ankle midline closure

The surgical technique was consistent in all cases. We obtained the Institutional Review Board (IRB) approval for the study. Patients underwent general anesthesia with a sciatic nerve catheter for postoperative pain management. Prophylactic antibiotics were administered intravenously, and a tourniquet was inflated to 250 mmHg. An anterior midline incision was made over the ankle. The ankle joint was approached through the interval between the extensor hallucis longus and the tibialis anterior tendons. The capsule was split and preserved for closure. All patients had a Scandinavian total ankle replacement (STAR Ankle; Stryker, Mahwah, NJ) TAA placed following the manufacture’s technique guide. A hemovac drain was placed deep into the capsule. The capsule, the extensor retinaculum, and finally the subcutaneous layer were closed with 3-0 Vicryl suture (Ethicon, Bridgewater, NJ). The skin was closed with a 3-0 nylon in a horizontal mattress technique (Figure 1).

**Figure 2:**
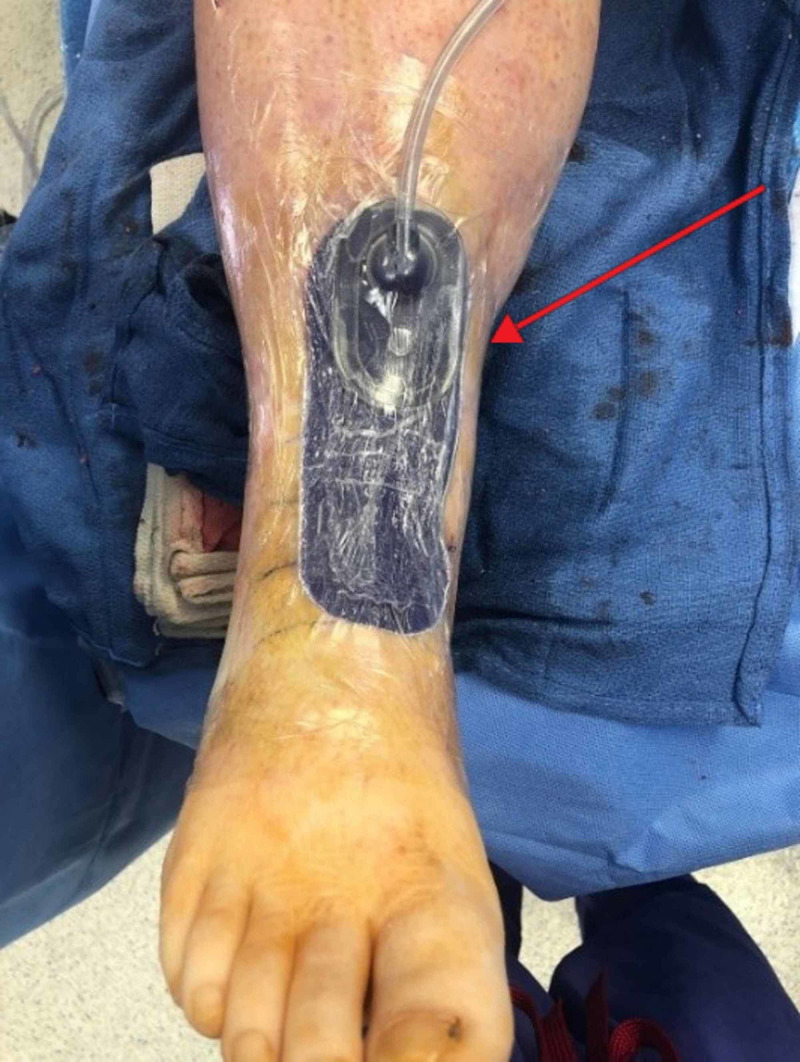
Application of PREVENA Incision Management System in surgery (arrow)

Prior to letting the tourniquet down, benzoin was placed around the incision. A 13 cm Peel & Place PREVENA dressing was applied according to manufacturer guidelines under sterile conditions at the conclusion of the operation and was connected to a PREVENA 125 Therapy Unit with a 45 mL canister at -125 mmHg continuous pressure. A positive seal was confirmed for each patient (Figure 2). The patient was placed into a bulky posterior sugar tong splint. The PREVENA remained in place until the postoperative office visit two weeks later. All patients were notified that the battery to the PREVENA 125 Therapy Unit would cease functioning after one week. The patients were asked to come in early for evaluation If they found the alarm on the device go off.

**Figure 3:**
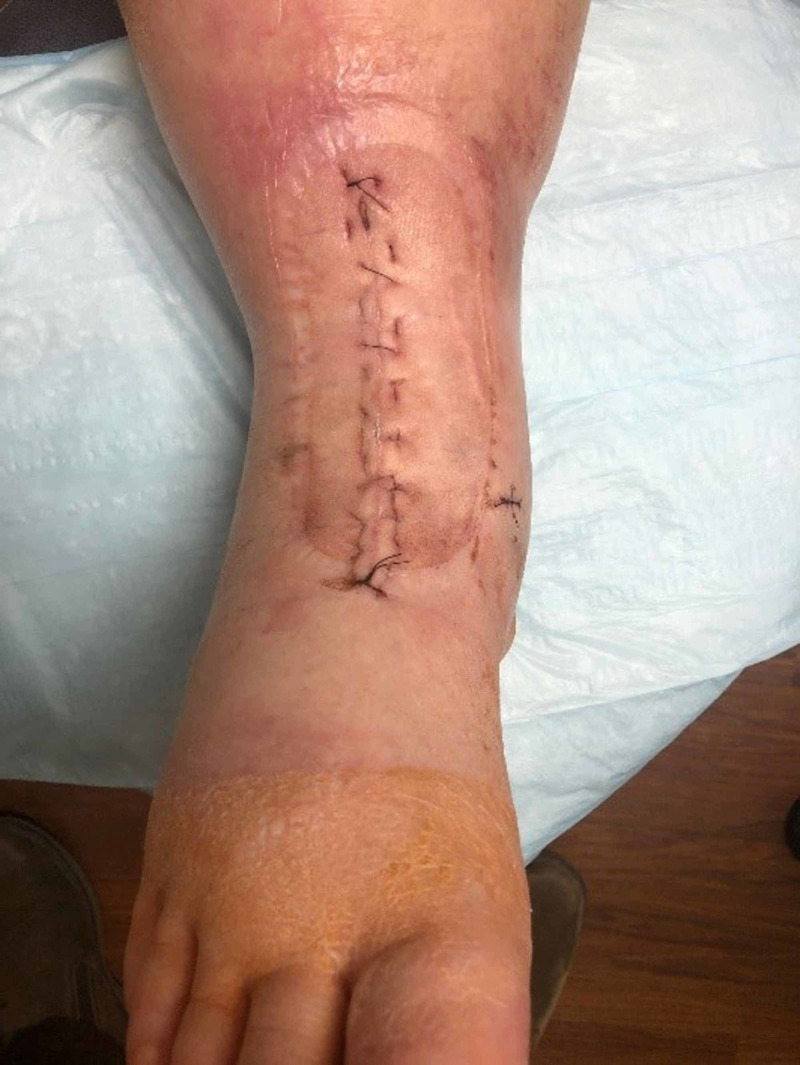
Removal of PREVENA at the two-week postoperative visit

**Figure 4:**
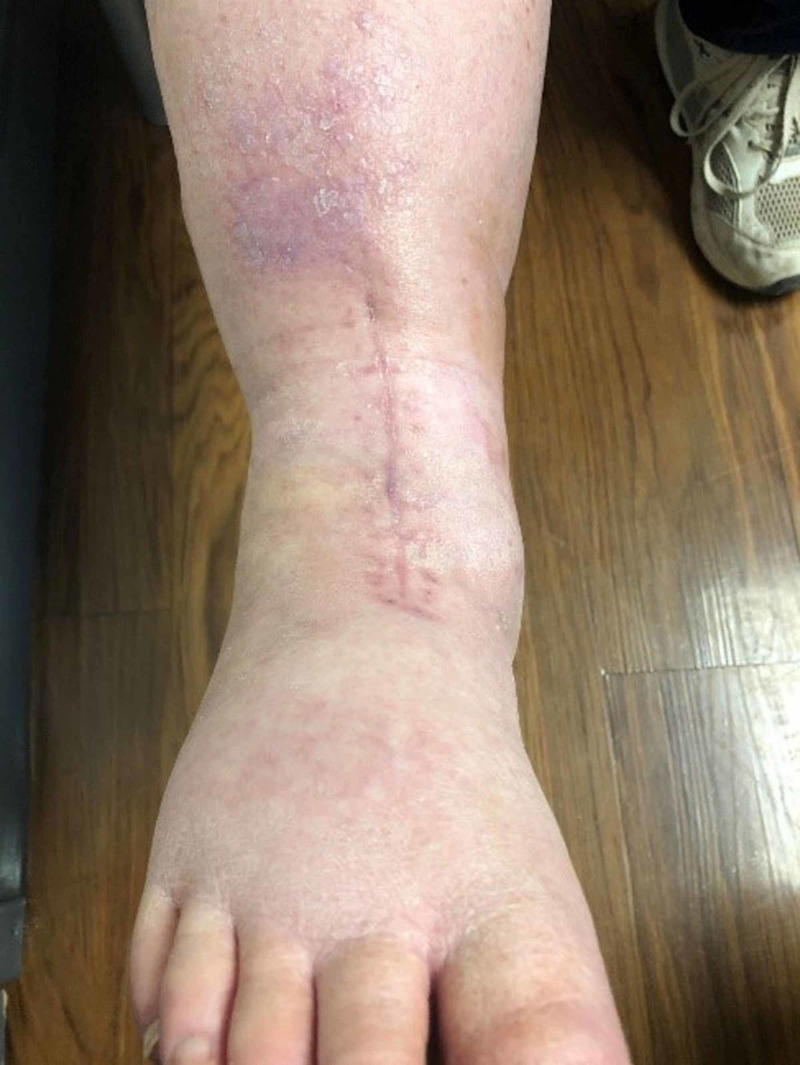
Incision site six weeks post-surgery

The splint, dressings, and PREVENA were removed when the patients presented for their first postoperative visit (Figure 3). If the incision looked well healed, sutures were removed. If there was any concern about the stability of the incision, the patient was brought back for suture removal the following week. Once sutures were removed, patients were placed into a postoperative boot, allowed to be partial weight-bearing, and advanced to full weight-bearing by four weeks. Physical therapy was started once the sutures were removed. Patients were weaned off the boot between six and eight weeks (Figure 4).

Data collection

Patients who underwent a TAA by a single surgeon and had a PREVENA placed at the time of surgery between 2015 and 2019 were identified. A retrospective chart review was performed for each patient. Demographic information along with factors shown to decrease wound healing, including diabetes, obesity, smoking, inflammatory arthritis, immunosuppressive medications, and hypertension, were recorded. Delays in the standard postoperative treatment due to incisional wound complications including infection or any complications related to the PREVENA were also recorded. An incisional wound was defined as any aspect of the incision that persisted beyond two weeks and requiring any supplemental wound care or debridement. Superficial infections included erythema and warmth consistent with cellulitis and increased wound drainage or purulence. Deep infections were defined as infections beneath the skin level that required surgical intervention.

## Results

Twenty-eight patients who had PREVENA ciNPT placed to the TAA anterior ankle incision were identified. The demographics and risk factors are illustrated in Table 1. At the time of the TAA procedure, the average age of the patients was 68 years (range: 53-89 years). Average BMI was 29.7kg/m^2^ (range: 23-42 kg/m^2^). Thirteen (45%) of the patients were obese according to the Centers for Disease Control and Prevention (CDC) criteria (BMI of ≥30 kg/m^2^) [12]. Two (7%) were smokers and 11 (38%) were former smokers. One (3.5%) patient had diabetes. Two (7%) patients had rheumatoid arthritis, and two (7%) were taking Humira (AbbVie, North Chicago, IL). Eighteen patients (62%) had hypertension.

**Table 1:** Demographic and risk factor data for total ankle arthroplasty patients with PREVENA BMI: body mass index

Variables	N	%
Number of patients	28	100
Average age (years)	68	
Average BMI (kg/m^2^)	29.7	
Obesity	13	45
Current smoker	2	7
Former smoker	11	38
Diabetes	1	3.5
Rheumatoid arthritis	2	7
Immunosuppressive medication	2	7
Hypertension	18	62
Adhesive allergy	1	3.5

No patient (0%) had incisional wound complications including incisional necrosis, excessive drainage, cellulitis, seromas, or hematomas requiring any intervention or dressing changes. Additionally, no patient (0%) developed superficial or deep SSIs (Table 2). All patients had their sutures removed at the planned first postoperative two-week visit. There was no delay in any of the patients' normal postoperative course.

**Table 2:** Results and complications related to use of PREVENA in total ankle arthroplasty patients

Complications	N	%
Superficial surgical site infection	0	0
Deep surgical site infection	0	0
Rash	1	3.5
Blistering	1	3.5
Seroma	0	0
Hematoma	0	0
Incisional dehiscence	0	0

None of the patients experienced device malfunction requiring the patient to come into the clinic for early evaluation. One patient (3.5%) listed an adhesive allergy (Table 1) and developed blisters where the adhesive was in contact with the peripheral skin away from the incision (Table 2). There was one (3.5%) patient who developed a rash to the peripheral skin (Table 2). Both patients' reactions resolved with the removal of the ciNPT and the surgical prep that was removed with soap and water.

## Discussion

To our knowledge, this is the first investigation evaluating healing rates and complications of the anterior incision used in TAA patients treated with PREVENA ciNPT. Our study demonstrated excellent results with no cases of wound complications or SSIs, and there was no postponement in the standard postoperative rehabilitation related to delays in incisional healing. Compared to historical data, PREVENA provided superior prevention of wound complications and SSIs. The rate of postoperative complications of TAA varies from 20-28%, with delayed wound healing, ranging from 4-28%, as the most cited complication [14].

**Figure 5:**
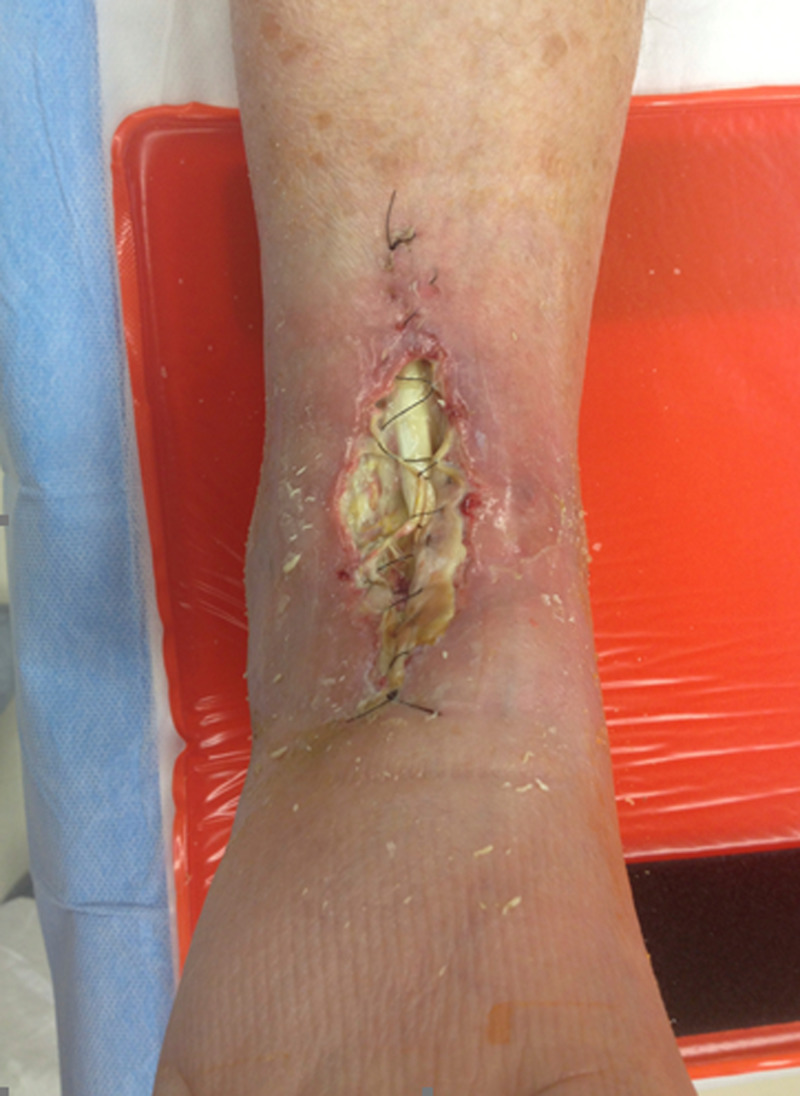
Example of dehisced total ankle arthroplasty incision with tibialis anterior tendon exposure

**Figure 6:**
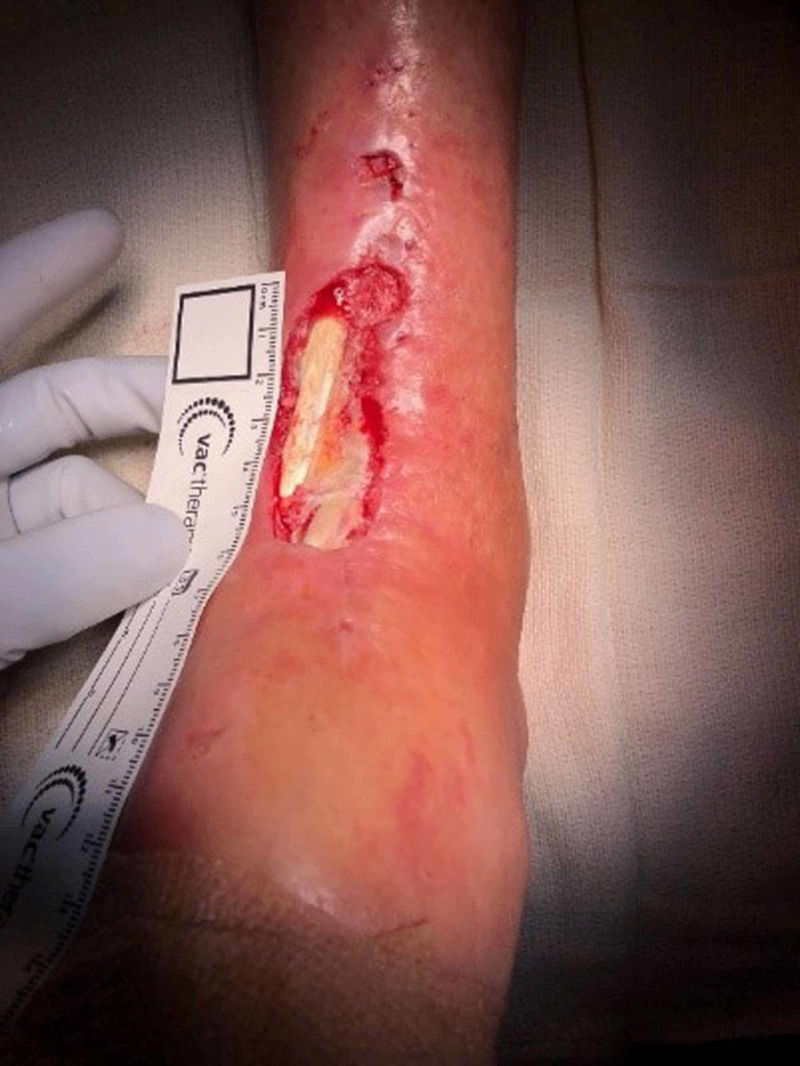
Example of total ankle arthroplasty surgical site infection with exposed tibialis anterior and extensor hallucis longus tendons

**Figure 7:**
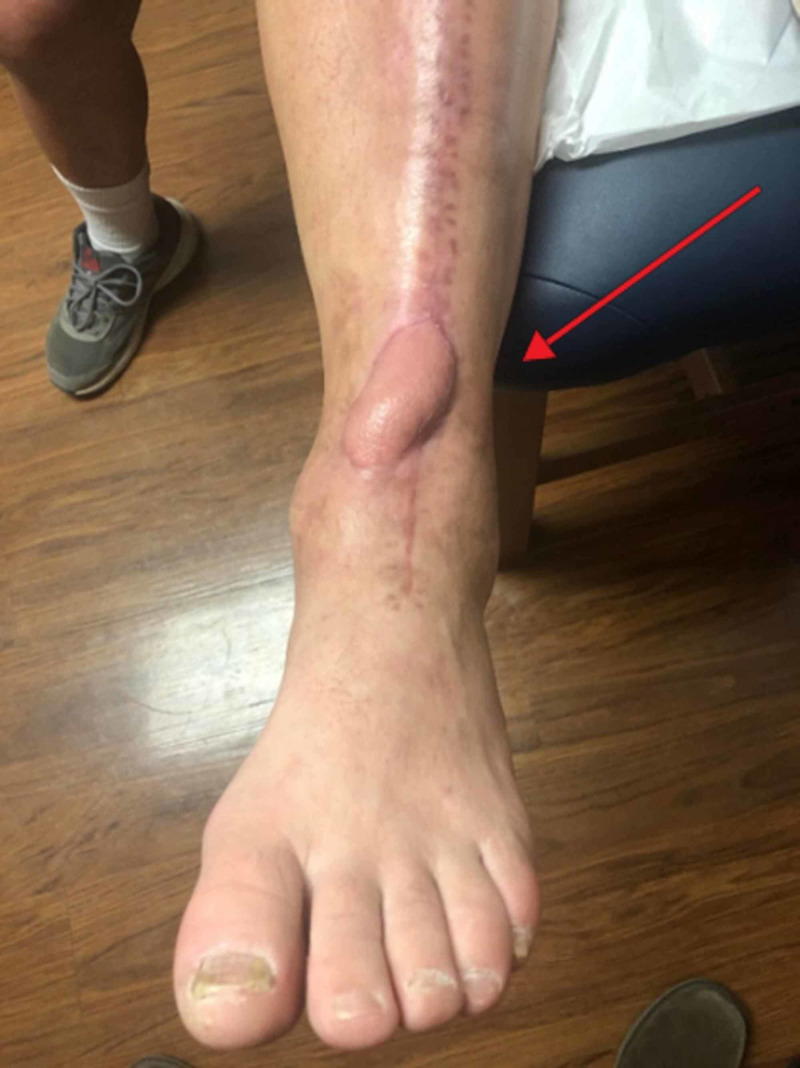
Example of free flap reconstruction following complications with original surgical incision of a total ankle arthroplasty (arrow)

The anterior midline incision from the distal portion of the leg to the dorsum of the foot is the most common surgical approach in TAA. This area has thin skin and limited soft-tissue coverage over the ankle joint. Wound complications with this incision can be significant because the prosthesis becomes exposed to bacteria relatively easily and soft-tissue coverage can be difficult. The impact of delayed healing or infection has a significant impact on implant success and longevity. Deep TAA infections were found to be associated with poor wound healing or wound drainage [15]. Patients with infections had a revision TAA rate of 23%. Therefore, if incisional site complications occur, they must be aggressively managed (Figures 5, 6) [6]. If the wound cannot be healed in a timely manner with the appropriate wound care, the patient might require reconstructive plastic surgery to correct the defect, and such complications could potentially require amputation (Figure 7).

Any peri-operative strategies to minimize postoperative complications and improve patient outcomes should be pursued. Preoperative optimization including smoking cessation, tight glycemic control, and weight loss can mitigate the likelihood of complications as well. The factors identified to increase the risk of wound complications and SSIs include diabetes, smoking, obesity, female sex, rheumatoid arthritis, and corticosteroid use [2,4,16]. Matsumoto and Parekh identified advanced age of 67 years and older and rheumatoid arthritis as factors associated with increased risk in TAA incisions, even with the use of ciNPT [11]. Obesity is associated with osteoarthritis of weight-bearing joints, and obese patients are more likely to request elective orthopedic surgery; this increases postoperative risks for wound and implant complications [17]. A study by Werner et al. has reported that 33% of patients undergoing TAA in 2011 were obese [17]. Our study population included patients considered to be at higher risk. These risks included an average age of 68 years, with two such patients having rheumatoid arthritis; moreover, 45% of our patients were considered obese based on the CDC criteria [12]. None of the patients in our cohort developed incisional dehiscence or SSIs, suggesting that PREVENA improves healing in patients with high-risk factors for wound healing complications. However, we do recommend that surgeons evaluate each patient individually to determine their likelihood for successful healing as we do not feel that using PREVENA will supersede appropriate surgical patient selection.

The standard practices of preoperative prophylactic systemic antibiotics, preoperative antiseptic, aseptic incision site preparation, and meticulous surgical technique are critically important. There are no standards for specific TAA postoperative care or dressing choice. Historically, dry dressings, casts, and splints have been used. It is fundamental that the reduction of incision pressure and efforts to reduce edema be addressed as swelling may delay wound healing by causing a decrease of blood flow to the surgical area [6]. Using sutures and staples introduces focal areas of reduced perfusion leading to ischemia and fibrosis to the surgical site [18]. Any additional therapy that would decrease swelling and tension and act as a physical barrier or prevent antimicrobial colonization would contribute to incisional healing.

PREVENA minimizes separation of the incision edges and realigns tensile strength along the incision instead of being concentrated along the individual sutures. This enables the skin edges to remain everted and leads to improved and often prompt healing of the incision. After the PREVENA application, the incision tension is decreased by about 45-70%, which approaches preoperative physiologic tension [7]. The reduction of the sheer stress along the suture line is key to maintaining the integrity of the closed incision [18]. This may be a result of the action of PREVENA to reduce excessive fluid in between the tissue layers. According to the PREVENA Incision Management System Product Monograph 2016, the reduction in excessive fluid allows for the lymphatic system to have improved function resulting in less edema and hence a smaller amount of tension along the incision. The additional benefit of facilitating the removal of fluid by negative pressure at a preset level of -125 mmHg continuous pressure helps to stimulate cell proliferation, decrease the inflammatory mechanism [19], increase oxygen saturation, and increase blood flow [6]. Our patients had excellent wound healing with minimal swelling at the incision site, and we did not identify any seroma or hematoma formation within this patient group (Figures 3, 4).

PREVENA may decrease infection mechanically and chemically. As an airtight seal, it prevents contamination from the outside to gain access to the incisional area. The addition of the 0.019% ionic silver layer that contacts the skin may be beneficial in reducing bacterial colonization that could occur with the traditional dressing in place and during the traditional dressing change. This could reduce the consumption of antibiotics and avoid contributing to antibiotic resistance [9,20].

Clinical outcomes have demonstrated good success with ciNPT. Using PREVENA in high-risk populations for total knee arthroplasty or total hip arthroplasty, investigators have found that after adjustment for inflammatory arthritis or periprosthetic joint infection, there was a decreased rate of wound complication to 5.4% compared to 17.5% in the control group that was treated with standard dressings [7]. There is limited data on therapies to improve healing and prevent or reduce SSIs of TAAs with an anterior incision. Using a PICO dressing, Matsumoto and Parekh demonstrated a 32% and 6% wound infection rate without and with ciNPT in TAAs with anterior incisions, respectively [11]. Our patient group developed no wound complications or SSIs using PREVENA (Figures 2, 3). This difference in complication rates between the above study and ours could be due to insufficient sample size or the result of a different comparative dressing choice. It is significant to note that PREVENA is the only ciNPT to have obtained the Food and Drug Administration (FDA) approval for reducing the incidence of SSIs in class I (clean) and class II (clean-contaminated) wounds according to KCI (a subsidiary of Acelity, San Antonio, TX) [21]. Also, the PICO dressing is a smaller dressing with a non-adjustable setting of -80 mmHg continuous pressure, which may translate to less potential regarding suction, reduction of tension, and reduction of edema to the skin.

A potential benefit of PREVENA to TAA patients is the reduction in scar formation, which may improve function through a better range of motion and tendon gliding. Since there is a paucity of subcutaneous tissue in the region of the TAA anterior incision, scars can lead to adhesion of tendons and prosthesis. Comparing ciNPT with standard wound care showed a reduction of scar thickness and width, increased tensile strength, and greater resistance to mechanical stress with increased collagen at the incision site. Placement over high-stress areas in the lower extremities where mechanical tension is involved in fibroblast movement during wound healing may act to reduce the formation of hypertrophic scarring [20]. These are important considerations, especially when planning the surgical site of TAA and for mitigation of the possible complications of TAA surgery [9].

In addition to being potentially devastating to the patient, wound complications and SSIs have a significant negative effect on healthcare costs. The patient may incur wound-care and home health-costs and expenses related to further imaging and labs, a possible return to surgery, further hospitalizations, and antibiotic therapy via oral or intravenous routes. There are estimated to be more than 20,000 surgical site arthroplasty infections annually after orthopedic surgery in the United States [22]. SSIs may increase a patient’s hospital stay by one or more weeks and increase direct costs by more than 300% [22]. The cost of ciNPT is estimated to be less than $500 for the therapy period. It is suggested that ciNPT is a cost-effective strategy to decrease complications and reduce SSIs [20]. With zero complications reported with 28 TAAs with the use of PREVENA, the economic burden appears significantly reduced, suggesting that ciNPT, and specifically PREVENA, may be a cost-effective measure to control wound complications postoperatively.

PREVENA was reliable in other ways too as there were no leaks or suction device problems. The only patient-related issue was skin reactions including rash and blistering to the adhesive around the dressing in two patients (7%). Both patients’ skin irritations resolved with no intervention other than washing the skin after the PREVENA was removed. One of these patients had a known history of reaction to adhesives. Therefore, we recommend that surgeons discuss signs and symptoms with patients prior to the placement of a ciNPT. Since ciNPT is designed to be in place for seven days, it would be worthwhile to study if the incorporation of adhesives approved for sensitive skin would limit allergic reactions that may lead to skin complications in an already fragile area of the anterior ankle.

The current study has some limitations, which include a low total number of patients, its retrospective case series design, and the lack of a control group for outcome comparison. However, based on the rates of possible wound problems and infections following TAA being as high as 28%, as well as a majority of our patients having at least one documented high-risk factor related to healing, we would have expected to see some wound complications in our cohort, which we did not identify. It is important to note that our data is not sufficient enough to compare the use of different types of ciNPT devices.

## Conclusions

TAA is a common procedure for the treatment of end-stage ankle arthritis. This procedure’s success is compromised when patients sustain a wound complication or SSI. Our study showed that the use of PREVENA reduces wound complications and SSIs in normal and high-risk patients undergoing TAA through an anterior approach. Device-related problems were rare, and patient issues were limited to skin reactions to the adhesive. Due to the relatively low cost of ciNPT and the high cost of treatment of wound complications and infections following arthroplasty, PREVENA appears to be a cost-effective adjunct to the surgical plan. Therefore, the authors recommend the use of PREVENA when performing TAA through an anterior incision.
